# Twenty Years Development of Tibial Cortex Transverse Transport Surgery in PR China

**DOI:** 10.1111/os.13214

**Published:** 2022-05-07

**Authors:** Zheng Liu, Chao Xu, Yi‐kang Yu, Dong‐peng Tu, Yi Peng, Bin Zhang

**Affiliations:** ^1^ The Second Clinical Medical College of Zhejiang Chinese Medical University Zhejiang China; ^2^ The Second Affiliated Hospital of Zhejiang Chinese Medical University Zhejiang China

**Keywords:** Ilizarov technique, improvement, microcirculation, tibial cortex transverse transport, wound healing

## Abstract

Tibial cortex transverse transport (TTT) surgery is an extension of the Ilizarov technique. Based on the law of tension‐stress, its primary function is to rebuild microcirculation which can relieve ischemic symptoms and promote wound healing. It has received more and more scholars' attention and has experienced a series of changes for 20 years since it entered PR China. The mechanisms involved have gradually become clear, such as the reconstruction of the polarization balance of macrophages, the promotion of vascular tissue regeneration, and the mobilization and regulation of bone marrow‐derived stem cells. TTT technique is mainly used in the treatment of chronic ischemic diseases of the lower extremities. It has recently been successfully used in the treatment of primary lymphedema of the lower extremities. A series of improvements have been made in the external fixator's style, the size of skin incision and osteotomy, and distraction method. For example, the annular external fixator has been redesigned as a unilateral external fixator, and accordion technology has been introduced. For distraction methods after surgery, there was no uniform standard in the past. The technique can also be used in combination with other treatments to achieve better effects, such as interventional therapy, negative pressure sealed drainage, 3D printing technology, traditional Chinese medicine. Nevertheless, the surgery may bring some complications, such as secondary fracture, nail infection, skin necrosis at the surgical site, etc. Reports of complications and doubts about the technique have made the TTT technique controversial. In 2020, the relevant expert consensus was published with treatment and management principles, which might guide the better application and development of this technique.

## Graphical abstract

The surgery of tibial transverse transport has shown definite efficacy in the treatment of chronic ischemic diseases of the lower extremities and may be gradually applied to the treatment of primary lymphedema in the future. With the gradual understanding of its mechanism of action, the continuous improvement of the technique, and the publication of relevant expert consensus, its clinical application will be further guided.

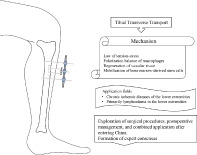

## Introduction

Tibial cortex transverse transport (TTT) is a continuation of the Ilizarov technique. Unlike the previous Ilizarov external fixation technique for longitudinal movement of the osteotomy segment, this operation involves lateral distraction of a bone chip on the tibial shaft. The purpose of TTT is not osteogenesis but local vascular tissue regeneration. Based on the law of stress‐tension, the continuous distraction of the tibial cortex chip can promote cell metabolism, accelerate tissue regeneration, rebuild microcirculation, and restore blood and oxygen supply to the lower limbs. It is now mainly used in the treatment of chronic ischemic diseases of the lower extremities^1^. Recently, TTT has been successfully used in the treatment of primary lymphedema in the lower limb^2^.

China's Qu *et al*.^3^ first applied TTT to the clinic in China. They published the article “The Therapy of Transverse Tibial Bone Transport and Vessel Regeneration Operation on Thromboangitis Obliterans” in 2001, which not only introduced TTT to PR China but also opened up the exploration of TTT by Chinese scholars. By 2021, the technique has been developed in PR China for a full 20 years. Its indications expanded from thromboangiitis obliterans to chronic ischemic diseases of the lower extremities, including diabetic foot ulcers, thromboangiitis obliterans, and arteriosclerotic obliterans, especially diabetic foot ulcers. Looking back at the past 20 years, TTT has been paid more and more attention, applied and studied by more and more scholars. As the use of the TTT has increased, reports of complications have increased. Many patients and even doctors have a hard time understanding how the technique works: patients with chronic ischemic diseases of the lower extremities have an insufficient blood supply and poor wound healing ability, and how can TTT surgery, an invasive treatment, achieve the purpose of restoring blood supply of lower extremities and promoting wound healing? Reports of complications and doubts about the technique have made it controversial.

In order to enable more people to know this technique, understand its mechanism of action, current application fields, and efficacy and safety, while at the same time allowing the technique to be better developed for clinical application, this paper reviews the 20‐year research progress of this technique since it was introduced into PR China.

## Methods

Three databases were searched, including PubMed, CNKI, and Wanfang databases. The literature about TTT published before March 2021 was searched with the following keywords: “lateral tibial transport,” “transverse tibial transport,” “transverse tibial movement,” “transverse bone transport,” “tibial bone immigration,” “tibial transverse transport,” “distraction osteogenesis,” “Ilizarov technique,” “Ilizarov bone transport,” and so on.

The inclusion criteria were: (i) content of literature is related to TTT surgery; (ii) articles can come from journals, dissertations, and conference reports, but the content must be complete; (iii) the types of articles can be randomized controlled studies, case reports, and reviews, etc.; and (iv) for clinical research articles, patients need to receive TTT surgery, which can be compared with conventional treatment or TTT alone, and the postoperative results must be reported.

The exclusion criteria: (i) low evidence level; (ii) unable to obtain full text; and (iii) the language of the text is neither Chinese nor English.

According to the inclusion criteria and exclusion criteria, two independent reviewers filtered those articles; a third reviewer would mediate when inconsistent judgment appeared. Subsequently, the qualified articles were carefully read to summarize the mechanism of action, application fields, improvement of surgical procedures in recent years, efficacy and safety, as well as the first expert consensus published recently. A total of 80 articles were eventually cited in the review.

## Results

### Mechanism of Action

#### Law of Tension‐Stress

Ilizarov, a doctor from the former Soviet Union, found that if the broken end of fracture and the long epiphyseal end is continuously and steadily distracted slowly, it can stimulate the regeneration of bone tissue, which is called “distraction osteogenesis”^4^. Further basic and clinical research led to forming the “law of tension‐stress”: a slow, steady, continuous distraction on living tissue stimulates local tissue regeneration and active growth. Ilizarov originally developed the TTT external fixator to thicken the bone and change its shape but unexpectedly found that the lower limbs' blood flow increased significantly^5^. Then, he performed TTT surgery on the dog's tibia, then distracted bone chip for 3 weeks and saw successful regeneration of the capillary network^6^. However, he did not conduct additional clinical research on the regeneration of vascular and tissue. Today, the goal of TTT is not intended to osteogenesis or change the shape of the tibia but to regenerate tissue, rebuild local microcirculation, and eventually restore blood and oxygen supply to the lower extremities^7^.

#### Reconstruction of Polarization Balance of Macrophages

Wound healing mainly includes three stages: inflammation, proliferation, and remodelling^8^, ^9^, ^10^, ^11^. Macrophages are the key to the transition from the inflammatory phase to the proliferative phase, and they can be divided into two types. M1 type macrophages can secrete various pro‐inflammatory factors to kill pathogenic microorganisms and control infection, but at the same time can cause damage to normal tissue. M2 type macrophages can secrete various anti‐inflammatory and growth factors to regulate immune response and promote repair of tissue damage^12^, ^13^. Gao *et al*.^14^ thought that the persistent existence of pathogenic microorganisms, which led to the macrophage polarization in the wound of severe diabetic foot ulcers, was unbalanced. Macrophages are mainly polarized into M1, while M2 is relatively deficient. Those may be the reason why the wound healing is prolonged. They observed tissue sections of wounds from patients after TTT and compared macrophage types, finding that the number of macrophages decreased 1 month after surgery. Meanwhile, the M1/M2 ratio decreased from 4.072 ± 0.502 before surgery to 3.098 ± 0.548 (*P* < 0.05) (Table 1). Therefore, they hypothesized that TTT could control the local wound inflammation, tend to make macrophages transform into M2 type, promote the reconstruction of macrophage polarization balance and anti‐inflammatory function, and finally heal the wound. There are limited studies on the reconstruction of the polarization balance of macrophages by TTT, and the specific role of TTT in this process can be further explored.

**TABLE 1:** Count of M1 and M2 macrophages and M1/M2 ratio in the severe diabetic foot before and after TTT surgery conducted by Gao *et al*.^14^

	Sample size	Pre‐surgery	1 month after TTT surgery	*P* value
Count of M1	10	156.270 ± 33.034	73.930 ± 15.065	<0.001
Count of M2	10	39.900 ± 11.120	28.950 ± 8.774	0.025
M1/M2	10	4.072 ± 0.502	3.098 ± 0.548	0.001

Abbreviations: M1, M1 type macrophages; M2, M2 type macrophages; M1/M2, the ratio of M1 to M2; TTT, tibial cortex transverse transport.

#### Promotion of the Regeneration of Vascular Tissue

**Fig. 1:**
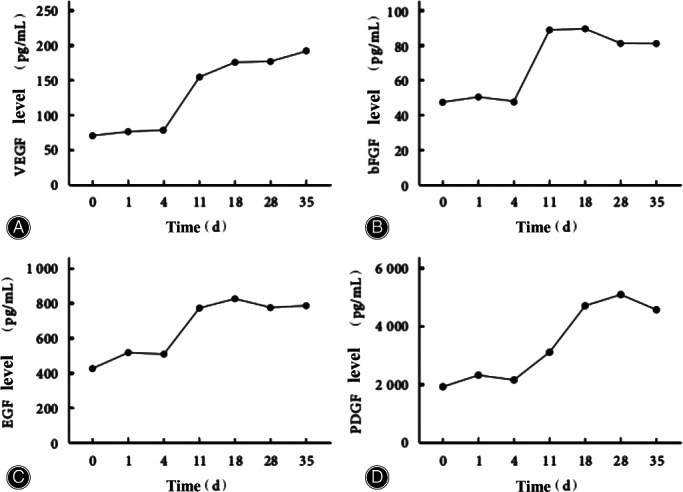
The expression level of angiogenesis‐related growth factors in the serum of patients before and after TTT surgery (0 day indicated for 1 day before the surgery) conducted by Ou *et al*.^22^ (A) The expression level of VEGF increased rapidly at 11 days after TTT surgery, and the expression level at 11, 18, 28, and 35 days were significantly higher than that before surgery (*P* < 0.05). (B) The expression level of bFGF increased rapidly at 11 days after TTT surgery, and the expression level at 11, 18, 28, and 35 days were significantly higher than that before surgery (*P* < 0.05). (C) The expression level of EGF increased rapidly at 11 days after TTT surgery, and the expression level at 11, 18, 28, and 35 days were significantly higher than that before surgery (*P* < 0.05). (D) The expression level of PDGF increased suddenly at 18 days after TTT surgery, and the expression level at 18, 28, and 35 days was significantly higher than that before surgery (*P* < 0.05). Source: Ou *et al*.^22^

TTT aims to rebuild the microcirculatory system and improve ischemia, so the wound is expected to heal with nutritional supply. Due to the long‐term inflammatory phase of the patient's wound, angiogenesis is difficult^15^. To facilitate the healing process, the critical issue to be addressed at this stage is angiogenesis. Lian *et al*.^16^ found that the expression levels of Ki‐67, platelet endothelial cell adhesion molecule‐1 (CD31), and vascular endothelial growth factor (VEGF) in marginal tissues of wound increased significantly 1 month after TTT surgery compared to pre‐surgery (Table 2). The increase in Ki‐61 reflects the enhancement of local cell proliferation. CD31 participates in signal transduction, promotes tissue angiogenesis, and maintains the integrity of vascular endothelial cells^17^, ^18^. VEGF can promote angiogenesis^19^, ^20^, ^21^. Therefore, they hypothesized that surgery could effectively promote cell proliferation and capillary regeneration in local tissues. Ou *et al*.^22^ studied the expression of angiogenesis‐related growth factors in patients' serum and reached a consistent conclusion. They revealed that VEGF, basic fibroblast growth factor (bFGF), and epidermal growth factor (EGF) expression levels were not significantly increased until the seventh day after the distraction and remained higher than preoperative levels until the end of distraction. Platelet‐derived growth factor (PDGF) expression level increased suddenly 2 weeks after the operation and remained high until the end of distraction (Figure 1). Both bFGF and EGF can promote cell proliferation and epithelialization of granulation tissue^23^. PDGF can promote debridement and repair^24^. This difference in expression level may be because distraction promotes vascular tissue regeneration in the initial stage and plays a repair role later. At present, observed growth factors are limited. Whether the expression levels of other kinds of growth factors also change due to TTT needs further study.

**TABLE 2:** The percentage of positive cells with Ki‐67, CD31, and VEGF staining in the wound edge tissue before and after TTT for severe diabetic foot conducted by Lian *et al*.^16^

	Sample size	Percentage of positive cell area		Z value	*P* value
		Pre‐surgery	1 month after TTT surgery		
Ki‐67	30	(1.850 ± 1.287) %	(7.480 ± 5.272) %	3.292	0.001
CD31	30	(0.395 ± 0.139) %	(1.082 ± 0.636) %	3.403	0.001
VEGF	30	(0.341 ± 0.217) %	(2.428 ± 1.502) %	3.780	0.000

Abbreviations: CD‐31, platelet endothelial cell adhesion molecule‐1; VEGF, vascular endothelial growth factor; TTT, tibial cortex transverse transport.

#### Mobilization and Regulation of Bone Marrow‐Derived Stem Cells

The osteotomy site is mainly in the middle and upper segment of the tibia, but the foot lesions and ulcers can heal after distraction. Hua *et al*. call this phenomenon the “summoning phenomenon”^25^. Some patients have ulcers in both lower extremities. After unilateral bone transport, contralateral ulcers, skin temperature, blood supply, and pain have also been influenced to a certain extent^26^, ^27^. These phenomena suggest that the effect of TTT may be systemic, which is similar to the mechanism of stem cell repair: stem cells can migrate to the site of injury, restore vascular endothelial function, promote vascular tissue regeneration, and heal wounds. Bone marrow is the largest stem cell bank in the body. Xiang^28^ found a significant increase in the number of hematopoietic stem cell colony formations in patients' serum 1 month after surgery (Table 3), which might be due to TTT mobilizing bone marrow‐derived stem cells. The expression level of the PI3K/ AKT signaling pathway was increased in patients' serum 1 month after surgery (Table 3), which might because TTT further activated the stromal cell‐derived factor 1 (SDF‐1)/ CXC chemokine receptor 4 (CXCR4) axis and PI3K/ AKT signaling pathway, as well as regulated the migration and functional differentiation of bone marrow‐derived stem cells to the injury site and promoted the repair and regeneration of skin, blood vessels, nerves, etc.

**TABLE 3:** Changes of hematopoietic stem cell colony formation, PI3K and AKT transcription 1 month after TTT operation and 1 day before TTT operation conducted by Xiang^28^

	Sample size	Pre‐surgery	1 month after TTT surgery	*t* value	*P* value
Count of BFU‐E	20	46.20 ± 7.43	74.95 ± 9.63	10.568	<0.05
Count of CFU‐GM	20	5.55 ± 1.54	10.60 ± 2.16	8.512	<0.05
Count of PI3K	20	1.00 ± 0.18	2.19 ± 0.51	10.365	0.000
Count of AKT	20	1.00 ± 0.21	2.27 ± 0.59	9.840	0.000

Abbreviations: AKT, protein kinase B; TTT, tibial cortex transverse transport; BFU‐E, burst forming unit erythroid; CFU‐GM, colony‐forming unit‐granulocyte, macrophage; PI3K, phosphatidylinositol 3‐kinase.

Lian^29^ found that the expression level of stem cell mobilization‐related chemokines in diabetic patients such as SDF‐1 was significantly up‐regulated 1 month after surgery compared with that before surgery (fold change:1.307113), suggesting that bone marrow‐derived stem cells were mobilized. It was also speculated that the continuous distraction caused by TTT would continuously stimulate and activate the SDF‐1/CXCR4 pathway, which could produce a long‐lasting effect. Vu Le Hoang Anh^30^ measured the SDF‐1 level in serum of patients before surgery, 1 month after surgery, and 3 months after surgery. The distraction period was 4 weeks. The SDF‐1 level in serum was significantly increased 1 month and 3 months after surgery than before surgery (Table 4). But the level of SDF‐1 in serum at 3 months after surgery was lower than that at 1 month after surgery. The difference of SDF‐1 expression levels in patients' serum at different stages has confirmed that the mobilization effect of bone marrow‐derived stem cells is indeed continuous.

**TABLE 4:** Changes of SDF‐1 in the treatment of severe diabetic foot with TTT surgery conducted by Vu Le Hoang Anh^30^

	Sample size	SDF‐1 (pg/ml)
Pre‐surgery	16	220.19 ± 16.72^a^
1 month after TTT surgery	12	368.46 ± 21.32
3 months after TTT surgery	10	318.22 ± 12.61

Abbreviation: SDF‐1, stromal cell‐derived factor‐1; TTT, tibial cortex transverse transport

^a^ Compared to other times, *P* < 0.05.

### Application Fields

#### Chronic Ischemic Diseases of the Lower Extremities

After TTT was introduced into PR China, it was first used by Qu *et al*.^3^ for the treatment of thromboangiitis obliterans, then by Wang *et al*.^31^ for the treatment of arteriosclerosis obliterans, and later widely used for the treatment of diabetic foot^32^, ^33^, ^34^. These diseases have different etiology, but their progression can lead to microcirculation disorders and chronic ischemic symptoms: claudication, rest pain, ischemic ulcers or gangrene, and in severe cases, amputation^15^, ^32^, ^35^. In the early stage, drugs can be used to dilate blood vessels, reduce lipids, and improve coagulation function. In severe cases, arterial access reconstruction, surgical debridement, and infection control can be performed^36^. However, these treatment methods' overall efficacy is not satisfactory, and the disease may occur repeatedly or even continue to progress, and the ulcers wound is difficult to heal. The TTT technique can improve ischemia, relieve pain, heal and repair wounds, and significantly improve the percentage of limb salvage by distraction. Therefore, it is mainly applied in the treatment of chronic ischemic diseases of lower extremities in PR China.

Besides, Yang *et al*.^37^ used this technique to treat 12 elderly patients with chronic foot ulcers, of which 11 patients healed and one patient's food was amputated. Nie *et al*.^38^ treated 42 cases of recalcitrant non‐diabetic leg ulcers, and compared with traditional treatment, TTT could facilitate the ulcer healing and reduce the healing time. It can be seen that TTT has a definite therapeutic effect on the wound caused by chronic ischemia.

#### Primary Lymphedema in the Lower Extremities

Lymphedema is a chronic disease. Primary lymphedema is caused by congenital disorders of lymphatic circulation, leading to symptoms such as pain, limb swelling, and skin tightness, etc. Complete decongestive therapy is a common form of physical therapy and basic treatment. However, the high technical requirements for physicians, nurses, and physical therapists make it challenging to apply this method. Surgical treatment, such as lymphatic‐lymphatic anastomosis or lymphatic‐venous anastomosis, can also be turned to, but surgery's difficulty is relatively great. No matter what kind of treatment is taken, the disease tends to relapse, and the cure rate is low^39^. Zhu *et al*.^2^ presumed that TTT, which can improve the blood circulation of limbs, could possibly improve the function of the lymphatic system, so they tried to use TTT to treat a patient with primary lymphedema of the right lower extremity who suffered edema, tight skin, and aching of the lower limb. The patient's pain was significantly relieved after surgery, and the lower extremity edema gradually subsided. There was no recurrence of edema during the 10‐month follow‐up after surgery.

TTT technique has no direct relationship with the lymphatic system, but it can improve the lymphatic circulation function and treat primary lymphedema. The mechanism remains unclear, and only one case has been reported so far. Whether this technique can be used as a novel method for treating primary lymphedema still needs more case studies and longer follow‐up to clarify long‐term efficacy. Meanwhile, it also means that there may be more potential TTT value that has not been discovered, which is worth exploring.

#### Improvements in Tibial Cortex Transverse Transport Technique

**Fig. 2:**
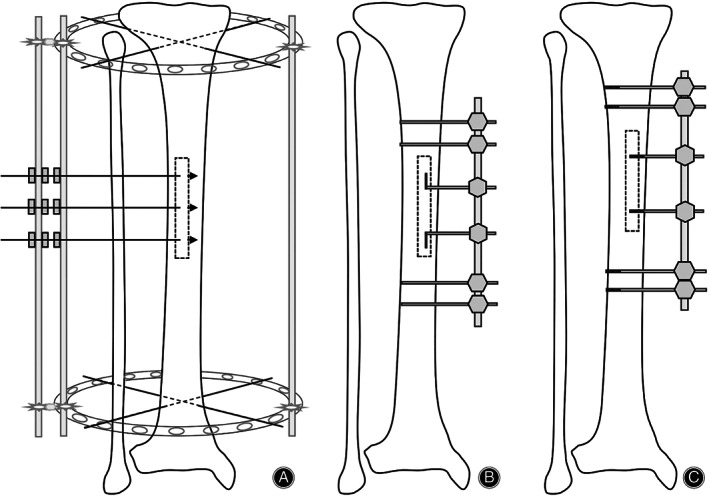
Changes in the external fixator and traction needles. (A) An annular external fixator with olive needles: these distraction needles can only distract the bone chip outwards. (B) A unilateral external fixator with curved steel needles: altered the osteotomy area into the medial side of the tibial crest, but these distraction needles can only distract the bone chip outwards. (C) A unilateral external fixator with threaded needles: these distraction needles can distract the bone chip in both directions.

When foreign scholars applied this technique, they used the traditional annular external fixator^40^, ^41^ (Figure 2A). Corticotomy was performed on the lateral surface of the tibia. Transverse incisions were 3 cm long, crossing the tibial crest, and mainly lateral. The longitudinal incisions were about 12 cm. The drill was used to drill holes, and then a bone knife was used to form a rectangular bone chip. Three olive wires equidistant to each other were used to pass through the rectangular piece's lateral cortex from the medial incision. The distal end of the olive wire was connected to Ilizarov external fixator with the slotted threaded rod. The distraction was started after 1 week: 1 mm per day till a distraction of 20 mm was achieved.

#### Exploration of Surgical Procedures

Qu *et al*.^3^ redesigned a unilateral external fixator when the TTT technique was applied for the first time in PR China (Figure 2B). They altered the osteotomy area into the medial side of the tibial crest and changed three olive wires into two curved steel needles. A 15‐cm longitudinal incision was made on the medial skin of the middle segment of the tibia. A pneumatic saw was used to penetrate the bone cortex to form a corticotomy window of 12 cm × 2 cm, and the bone marrow should be protected during this process. Two curved steel needles passed through the cortex and then assembled the external fixator. The distraction was started after 5 days: 1 mm per day, divided 15–20 times, till a distraction of 22 mm was achieved. The unilateral fixator is smaller than the annular fixator and thus can reduce trauma. Some scholars thought that the advantages of selecting corticotomy on the tibia's anteromedial surface were that the medial approach could avoid injury to the common peroneal nerve and the flat surface here was easier for corticotomy^42^.

In reports of Hua *et al*., they selected the middle and upper segment of the tibia for cortical osteotomy, where the circumference is longer, to reduce the risk of fracture^32^, ^43^, ^44^. The periosteum was cut longitudinally and opened entirely to both sides to expose the corticotomy area. A pneumatic saw was used to form a corticotomy window. Two 2‐mm threaded needles were used for distraction (Figure 2C). The distraction was started after 5 days: 1 mm per day, divided 4 times, for 21 days, and then the bone chip was moved back in place at the same speed (accordion technology). External fixation was kept for 4 weeks, and the external fixator could be removed if callus formation was found on reexamination radiographs.

The medial‐then‐lateral movement of a bone chip, just like the use of an accordion, is called accordion technology. This non‐invasive technique is based on the law of tension‐stress^45^ and does not require injecting any drugs, proteins, or growth factors^46^. Before accordion technology was introduced into TTT surgery, it has been applied to deal with delayed union or nonunion after treatment of bone defect or infection with Ilizarov external fixation. Studies have proven that compression, distraction, and recompression of the broken end of fracture can regulate the growth factors and signaling pathways related to osteogenesis in the human body, thus stimulating the formation of microcirculation and promoting bone healing^45^. Introducing accordion technology into the TTT technique has the following advantages: the combination of median and lateral transport patterns can return the bone chip to its original position, avoiding humps on the surface of the tibia, and prolong the distraction time persistently stimulating the formation of microcirculation. Hua *et al*. utilized threaded needles that allowed the bone chip to be moved in the opposite direction enabling “accordion technology” and made it easy to remove. So, this is a significant innovation.

**Fig. 3:**
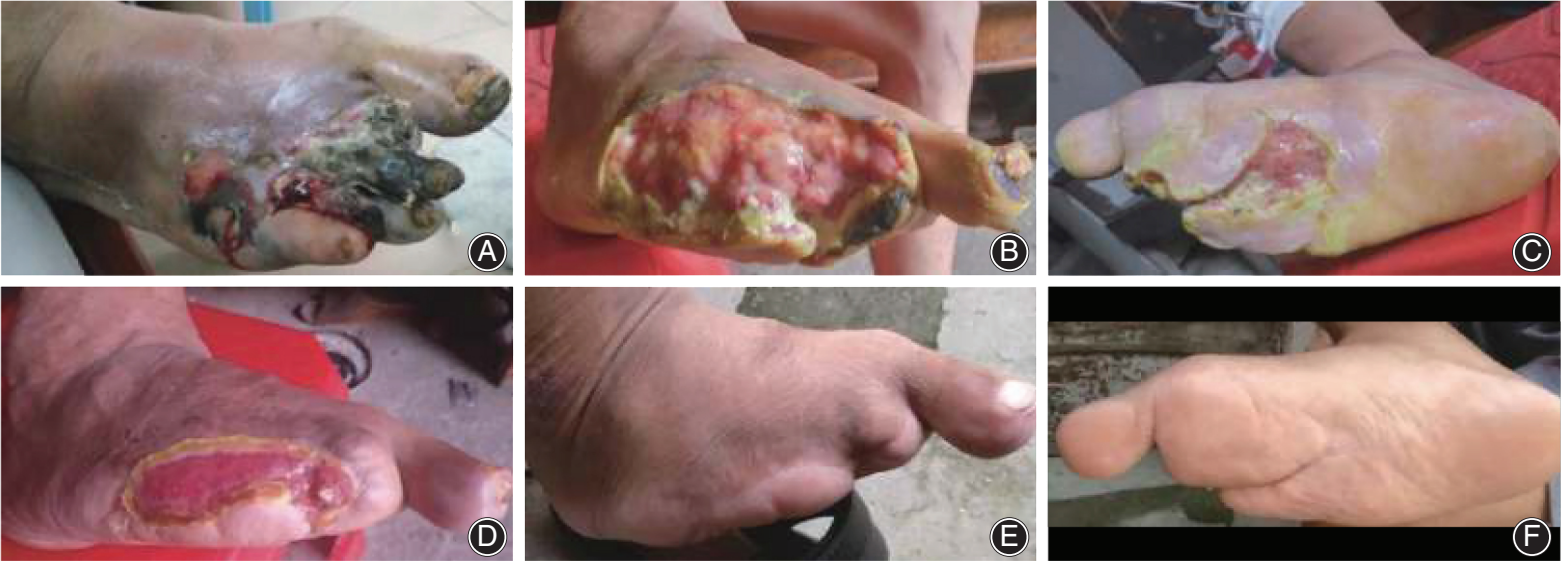
A 49‐year‐old male patient with diabetic foot (Wagner 4) and uremia treated by Hua *et al*.^25^ These photographs are cited from their research. (A) Preoperative wound. (B, C) The wound after debridement. (D) The wound at 8 weeks after TTT: the wound was much narrower, and the granulation tissue was healthy. (E, F) The wound at 1 year after TTT: the wound completely closed. Source: Hua *et al*.^25^

The medial tibia's skin tissue is thin, and the blood supply is insufficient, so the wound is difficult to recover and susceptible to infection. Researchers have been trying to make skin incisions or corticotomy sizes smaller (Figure 3). Yang *et al*.^47^ shortened the incision to 8–10 cm, and the corticotomy size was still 10 cm × 2 cm. Yang *et al*.^48^ improved the surgical incision to three smaller incisions with a distance of 2 cm and a length of 1.5 cm, while Ou *et al*.^49^ used the same method, but the incision size was 3 cm. They both kept the size of the corticotomy unchanged. In this way, the surgical trauma was reduced while the surgical field exposed, thereby reducing the occurrence of adverse complications. From another perspective, Cen *et al*.^26^ reduced the incision of the medial tibia to 3 cm and then used a microporous osteotomy apparatus to make a 5 cm × 1.5 cm bone chip. Both the incision and osteotomy areas were reduced, but the clinical efficacy was not affected.

Vu Le Hoang Anh^30^ reported that two curved incisions of 2.5 cm were made with a 3 cm medial interval in the middle of the tibia. Bone windows of 3.5 cm × 1.5 cm were made at an interval of 2 cm below the two incisions, respectively, which was called “日”‐type osteotomy. A 4‐mm traction needle was screwed into each of the two bone chips to distract them. A review of related studies shows that “日”‐type osteotomy is less commonly used. Simply reducing the osteotomy area can still obtain a good effect. In contrast, the “日”‐type osteotomy is complicated, increasing the trauma and prolonging the operation time. Maybe that is why 5 cm × 1.5 cm bone windows are preferred clinically.

When Qu *et al*.^3^ first used this technique in PR China, they emphasized that bone marrow should be protected during corticotomy so as not to affect the regeneration of intramedullary tissue. Later, Cheng *et al*.^50^ added that the periosteum should also be protected. Jia *et al*.^51^ used “门”‐type window opening to retain the periosteum of the fibula side and then opened the periosteum and inserted the needles. It has been reported that the periosteum can promote the regeneration of the local microvascular network^52^. The animal experiments conducted by Cao *et al*.^53^ confirmed that compared with the traditional “rectangular” window opening, the “门”‐type window opening significantly increased the peripheral blood vessel area and the percentage of vascular endothelial cells was significantly higher. So, the periosteum protection can provide a suitable environment for the subsequent regeneration of the microvasculature network.

#### Exploration of Postoperative Management

There is much variability in how long it takes to start distraction after surgery. Li *et al*.^54^ began on the second day after surgery. Zhang *et al*.^55^ took X‐rays for reexamination 5–7 days after the operation and started distraction according to the wound condition. Some researchers^56^, ^57^, ^58^ waited at least 1 week after surgery before starting. Qu *et al*.^3^ advocated starting the distraction at least 5 days after surgery. Jia *et al*.^51^ thought that the possible explanations were as followed: First of all, as the soft tissue on the inner side of the tibia is thin and the blood supply is insufficient, performing the distraction immediately after surgery is unfavorable for healing. Secondly, delayed movement is helpful to the healing of the periosteum. Then, the formation of the tibial bone window will reduce the intramedullary pressure, leading to the release of local microvascular spasm and increased bleeding, so early movement may increase bleeding. Last but not least, delayed movement avoids pain in the surgical area. Although the timing of the onset of distraction is different in the current study, no randomized controlled clinical study has determined this factor's effect on efficacy.

There was also no uniform standard for the speed and duration of distraction. At first, Qu *et al*.^3^ used 1 mm per day, divided 15–20 times, till a distraction of 22 mm was achieved. Hua *et al*.^32^ introduced the “accordion technology” and distracted 1 mm per day, divided 4 times, medial transport for 3 weeks and then lateral transport for 3 weeks. Besides, 1 mm per day, divided 4 times, for 10 days, and then moved back^59^ 1 mm per day, divided 5–8 times, for 2 weeks, and then moved back^57^. Medial transported for 7 days and lateral transported for 7 days, then repeat medial transported for 7 days and lateral transported for 7 days^60^. Sun *et al*.^61^ started distraction 1 mm per day, divided 3–4 times. If the patient had pain or other discomforts, the distraction should be suspended for 1–2 days or changed to 0.5 mm per day, and the duration of the whole transport was 14–16 days. Ye *et al*.^62^ also pointed out that transport speed and distance should be determined according to the skin condition. As for how long the distraction lasts, at what rate, and how many rounds of distraction can bring the most excellent benefits, the current selection is mainly based on experience. There is a lack of relevant research, which is worth exploring.

#### Combined Application of Tibial Cortex Transverse Transport Technique

Wang *et al*.^63^ indicated that TTT could only rebuild the microcirculation system from the perspective of vascular surgery. It is not easy to achieve the expected effect if the large and middle arteries cannot be guaranteed to have sufficient blood flow. The limitation of the TTT technique prompted scholars to break through the restriction by combining other technologies, which achieved satisfactory results.

For example, femoral‐femoral artery bypass grafting combined with TTT^58^, or interventional therapy combined with TTT^64^, was used for severe vascular occlusion in the lower extremities. These treatments unclog the blocked blood vessels, restore blood supply to the lower extremities, and restore microcirculation. Negative pressure sealed drainage^51^, ^65^, ^66^, or antibiotic‐embedded bone cement^67^ combined with TTT can control infection and increase local blood supply, jointly promoting wound healing. The 3D printing technology is used to make individualized surgical guide plates, which can accurately point and orient in operation according to the planned digging range, needle insertion position, and angle, reducing operation time and blood loss^57^. Autologous platelet‐rich plasma combined with TTT can effectively shorten the wound healing time and reduce complications^68^, ^69^, ^70^. Platelet‐rich plasma is an emerging technique in recent years. Although it has been reported to have specific effects, it has a high cost and requires repeated use during treatment. Whether it can bring exact benefits to patients remains to be studied. Some traditional Chinese medicines can also be used after surgery, which may be an excellent complementary and intensive treatment for TTT^71^, ^72^. Traditional Chinese medicine treatment based on syndrome differentiation has unique advantages, relatively low cost, and high patient acceptance, worthy of clinical research.

### Clinical Efficacy and Safety

Xu *et al*.^73^ reported that TTT was used to treat 35 cases of thromboangiitis obliterans. Thirty‐two patients successfully regenerated the blood vessels, their rest pain and intermittent claudication were significantly relieved. Histopathological examination performed on two of the 32 effective patients 2 months after surgery showed that the vascular endothelial cells in the vascular regeneration area were actively dividing and proliferating. Many capillaries and arterioles were born with the lumen unobstructed. Angiography performed 3 months after surgery in 12 patients showed a web of new arterioles emerging from the distraction area to the foot. Skin necrosis occurred in two cases, and nail infection occurred in four cases, which were cured after symptomatic treatment. Hu *et al*.^74^ treated 66 cases of thromboangiitis obliterans, and the skin temperature and blood oxygen saturation of the toe on the operated side increased significantly 2 months after surgery. Yang *et al*.^48^ treated 52 cases of thromboangiitis obliterans, and after 2–3 years of follow‐up, 45 cases of ulcers and wounds were successfully healed, with a total effective rate of 87%. Seven cases were amputated due to skin necrosis at the surgical incision or aggravation of ischemia, including five cases of popliteal artery embolization and two cases of posterior tibial artery embolization. The reason for the failure of these cases is that microcirculation is severely impaired, and it is difficult to regenerate. Therefore, the patency of the popliteal artery is essential for successful bone transport.

**Fig. 4:**
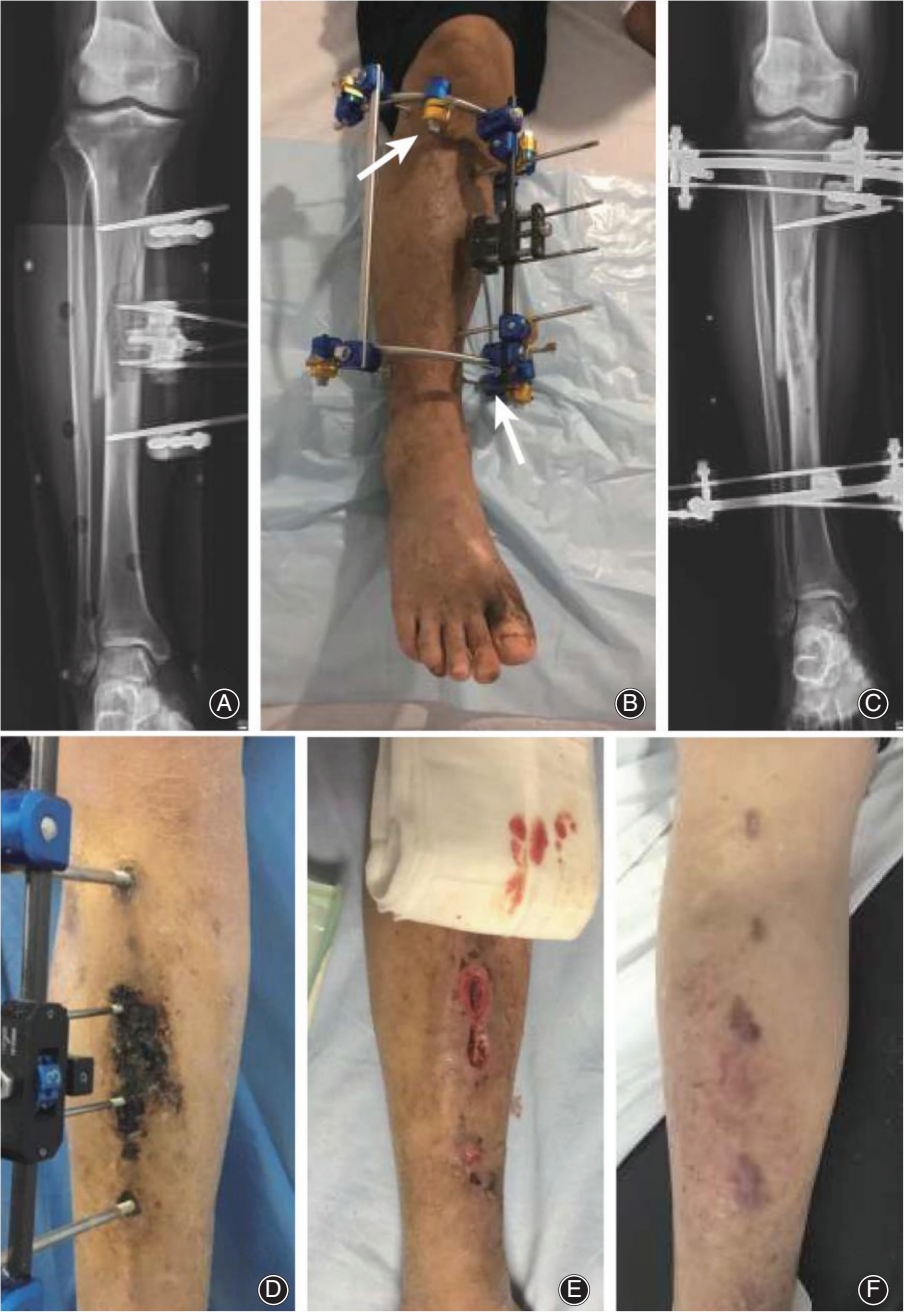
Complications after TTT surgery reported by Zhang *et al*.^76^ These photographs are cited from their research. (A–C) A 64‐year‐old male patient with tibial shaft fracture at 3 weeks after TTT surgery. (A) Fracture after TTT surgery. (B) Dealing with fixing pin (arrow) to strengthen external fixation. (C) Fracture healing at 2 months after tibial shaft fracture. (D–F) A 71‐year‐old female patient with skin necrosis in osteotomy area at 32 days after TTT surgery. (D) The skin necrosis in the osteotomy area. (E) The external fixator was removed, and debridement was performed. (F) The wound healed 1 month later. Source: Zhang *et al*.^77^

**Fig. 5:**
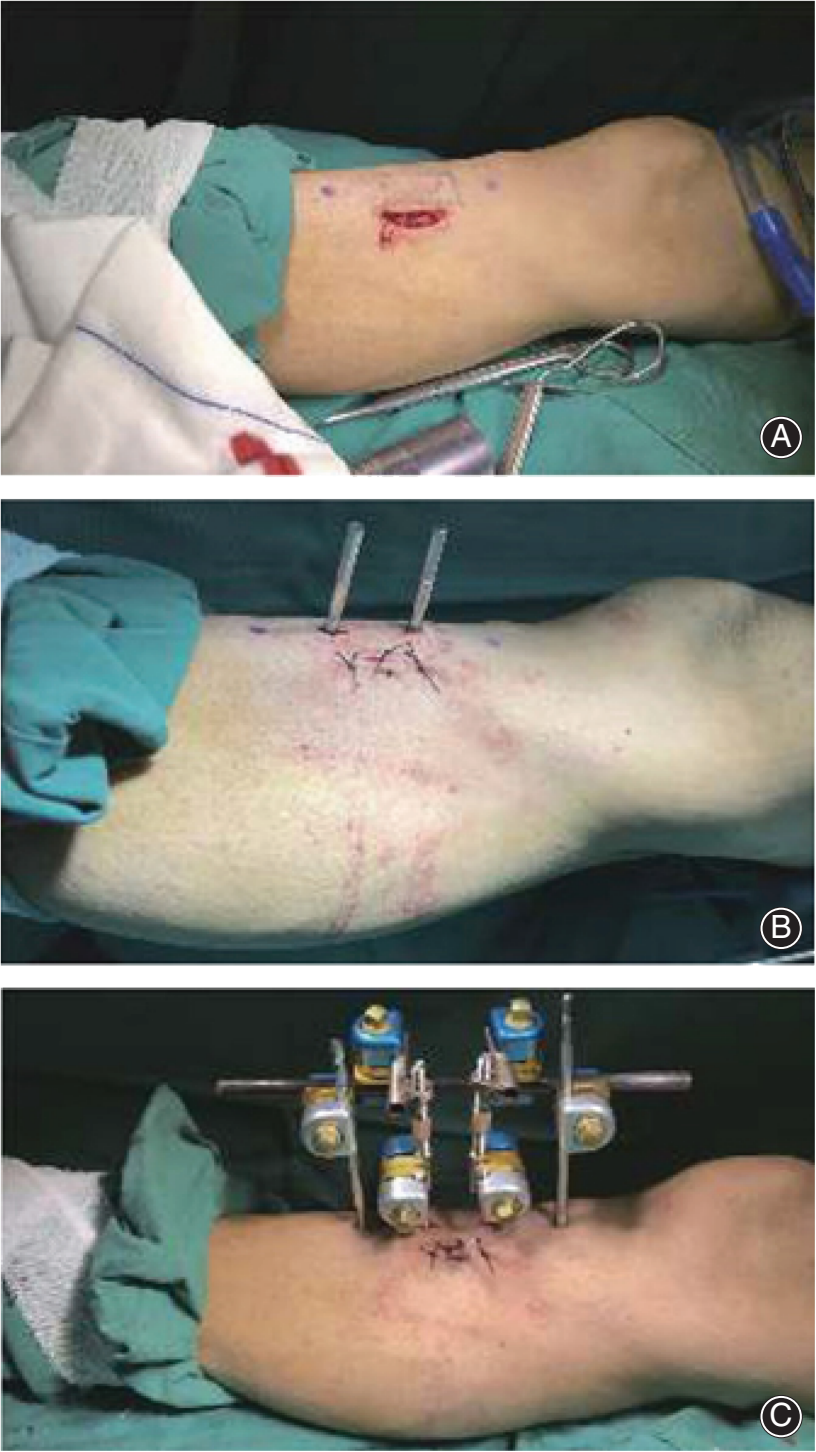
Steps of minimally invasive TTT surgery by Hua *et al*.^25^. These photographs are cited from their research. (A) The location of the osteotomy was planned preoperatively, and then a 3‐cm arc incision was made. (B) After the bone chip (5 cm × 1.5 cm) was cut with a miniature osteotomy device, and two threaded needles were inserted. (C) The incision was sutured, two needles were inserted into the tibia to stabilize the external fixator. The operation was completed. Source: Hua *et al*.^25^

Hua *et al*. has used the TTT technique to treat diabetes since 2013. There have been more than 516 patients with an average follow‐up of 32 months, and the limb salvage rate is 96.1% (Figure 4). One hundred and thirty‐six cases were followed up for 2 years. Four cases were amputated. Four cases had a relapse, and two cases had recrudescence^25^. Tian *et al*.^75^ conducted a meta‐analysis on the efficacy and safety of the TTT technique in treating diabetic feet. Compared with conventional treatment, TTT can significantly relieve pain, increase skin temperature, shorten ulcer healing time, improve the healing rate, and reduce amputation and recurrence rate. Common postoperative complications include tibial fracture, nail infection, non‐healing wound, tibial deformity, osteomyelitis caused by the wrong move of the bone chip, etc. However, the incidence of complications is low, showing reliable safety. It should be pointed out that the number of pieces of literature included in this study was limited, so the conclusions need to be confirmed by more clinical studies. Zhang *et al*.^76^ retrospectively analyzed 196 cases of diabetic foot treated by TTT, and 41 patients developed complications. Among them, there were 18 cases of nail infection, 12 cases of skin necrosis in the corticotomy area, and nine cases of tibial shaft fracture, all of which healed after symptomatic treatment (Figure 5). Two patients underwent amputation due to persistent foot infection and gangrene. Qin *et al*.^77^ applied this technique alone in the treatment of 19 patients with diabetic foot. Their pain was significantly relieved after the operation, but the blood circulation and nerve function were not significantly improved. The ulcer's healing rate was only 47.4%, and there were two cases of recurrence, which was quite different from most reported results. Fan *et al*.^78^ also pointed out that the function of TTT should not be exaggerated, and the complications brought by surgery should not be ignored. TTT alone cannot achieve the therapeutic purpose entirely, and debridement, wound care, anti‐infection, and other measures are also of great significance. (The clinical efficacy and complications of some studies are shown in Appendix Table A1.)

### Formation of Expert Consensus on the Tibial Cortex Transverse Transport Technique

TTT technique was born at the end of the last century and has not been widely used globally yet. Chinese researchers have been exploring it for two decades and have gained some experience in the treatment. Simultaneously, the technique has been doubted because of its complications and the fact that it is entirely different from traditional therapies. After 20 years of development, this technique has proved to be an effective way to treat chronic ischemic diseases in the lower extremities, especially in the treatment of the diabetic foot. The Chinese Association of Orthopaedic Surgeons, Taskforce Group of Tibial Cortex Transverse Transport Technique for the Treatment of Diabetic Foot Ulcers was established in 2019. Together with experts in various fields, they published “Expert Consensus on the Treatment of Diabetic Foot Ulcers Using Tibial Transverse Transport (2020)” in 2020^79^, which proposed the following treatment and management principles.

No tourniquet is applied after anesthesia to protect blood supply to the lower extremity. The corticotomy site shall be 5 cm below the tibial tuberosity on the medial tibia. A 3‐5‐cm arc incision is made, or multiple 1‐cm incisions perpendicular to the outer edge of the corticotomy area. An osteotomy device is used to make a 5.0 cm × 1.5–2.0‐cm bone chip without dissecting the periosteum. Then two 2.5‐mm distraction needles and two 4.5‐mm fixation needles are used to mount the external fixator. Five days after surgery, transversely distract the bone chip outward at a speed of 1 mm per day, divided 2 times, for 2 weeks. Stop moving for 3 days, and then compress the bone chip back at the same speed. X‐ray shall be taken 7 days after distraction to check the position of the bone chip. When the whole distraction is done, take another X‐ray film to make sure the bone chip is back to the correct position. If the wound healing is slow, it can be distracted repeatedly. If the wound is recovered, remove the external fixator directly and protect the operated limb with small splints or braces for 6–8 weeks till the corticotomy has healed on an X‐ray film.

Further simplification of the external fixator can reduce the risk of complications and reduce the impact on the patient's daily life. Surgery without using a limb tourniquet can protect the blood supply to the lower extremity to some extent. Reducing the incision and osteotomy area, emphasizing the protection of the periosteum, and using an osteotomy device to make the bone chip can reduce the damage to bone and local tissue. These improvements will not significantly increase the difficulty of surgery but contribute to wound healing and microcirculation reconstruction. It is a new method to use splints or braces after the external fixator is removed. Yang *et al*.^80^ implemented this method and asked patients to practice partial weight‐bearing walking of the operated limb. The patient needed to be reexamined monthly, and the limb could be fully weight‐bearing after the bone chip had healed completely. Among the 20 patients with thromboangiitis obliterans, one case was amputated due to foot ischemia aggravation, and one case was amputated due to bone chip infection. No tibia fracture or other complications occurred in the rest of the patients.

### Summary and Prospects

TTT technique has been used in PR China for 20 years, and the past 20 years have witnessed the transformation of this technique. It has a particular effect on the treatment of lower limb chronic ischemic diseases by reconstructing the microcirculation system. There may also be potential roles that have not yet been discovered. Due to its simple operation and short learning curve, this technique has been applied by more and more scholars. The lack of uniform standards in the past has led to differences in application, management, and even efficacy. Reports of complications have cast doubt on this invasive treatment and made it difficult for doctors and patients to understand and accept.

The publication of the “Expert Consensus on the Treatment of Diabetic Foot Ulcers Using Tibial Transverse Transport (2020)” is another milestone for the TTT technique. It will further guide the clinical application in the future so that the conclusions provided will be more reliable. At the same time, we should not just focus on the efficacy of this technique while ignoring its complications. We need to fully communicate with patients to get their understanding and cooperation so that they can have reasonable expectations and high compliance and carry out treatment under the cooperation of both doctors and patients. In the future, the mechanism of action of this technique is still worth further study, and the application fields are worth further exploration. It is also hoped that more combined therapies will bring new hope to more patients suffering from pain.

APPENDIX

**TABLE A1:** The efficacy and complications of the TTT technique

Author	Disease	Sample size	Results	Efficacy	Amputation	Complications	Follow‐up
Qu^5^	Thromboangiitis obliterans	18	Skin temperature increased, numbness disappeared, symptoms of intermittent claudication relieved, and angiography revealed an extremely rich network of blood vessels around the bone chip in the distal leg.	18 (100%)	0	No description	3 months
Hua *et al*.^25^	Diabetic foot	516	Rest pain relieved and blood flow to the feet increased significantly.	496 (96.12%)	20	Incision infection or skin necrosis (7) Secondary fracture (6) Acute arterial embolism (2)	7–72 months 11 cases recurred
Cen *et al*.^26^	Diabetic foot	130	Skin temperature increased, VAS score decreased, histological examination showed a decrease in inflammatory cells and an increase in myocytes, CT angiography showed a significant increase in collateral circulation.	124 (95.38%)	2	Wound nonhealing (6)	4–34 months
Yang *et al*.^37^	Chronic ulcers in the elderly	12	Skin temperature increased, the ankle‐brachial index improved, and CRP, ESR, and WBC indexes decreased.	11 (91.67%)	1	No description	/
Yang *et al*.^47^	Diabetic foot	8	Skin temperature increased, VAS score decreased, CT angiography in the operated limb showed that the dorsal foot artery was clear, the calf artery was open, the collateral artery was increased, the blood flow rate was faster, and the circulation was improved.	8 (100%)	0	0	1–3 months
Yang *et al*.^48^	Thromboangiitis obliterans	52	Skin temperature increased, VAS score decreased, the ulcers healed, angiography revealed many new small arteries in the distal tibia, interwoven into a network and extended laterally and diagonally to the muscle and subcutaneous tissue.	45 (86.54%)	7	Massive cerebral embolism (1) Nail infection (3) Deep vein thrombosis in lower limbs (1)	2–3 years Three patients recurred and underwent surgical treatment again
Ou *et al*.^49^	Diabetic foot	23	Skin temperature increased, VAS score decreased, Barthel index score increased.	21 (91.30%)	1	Fat liquefied at the incision (3)	12–19 months
Cheng *et al*.^50^	Diabetic foot Thromboangiitis obliterans	21	Rest pain and peripheral neuropathy relieved, skin temperature increased, the ulcers healed, WBC count and erythrocyte sedimentation rate decreased.	20 (95.24%)	1	Surgical site infection (1)	3–15 months
Jia *et al*.^51^	Diabetic foot	19	The ankle‐brachial index increased, Michigan Neuropathy Screening Instrument (MNSI) decreased, angiography or vascular ultrasound demonstrates microvascular network regeneration in the affected feet.	17 (89.47%)	0	Nail infection (2) Secondary fracture (2) Severe pneumonia (1)	3–13 months
Li *et al*.^54^	Diabetic foot	21	The ankle‐brachial index increased, the ulcers healed, and vascular ultrasound found increased blood flow in the affected feet' anterior and posterior tibial arteries.	21 (100%)	0	Severe ulcer infection on the contralateral foot (1)	13–24 months One patient died 13 months after surgery due to systemic sepsis due to severe ulcer infection on the contralateral foot
Zhang *et al*.^55^	Diabetic foot	10	Resting pain relieved, skin temperature increased, the ulcers healed.	10 (100%)	0	0	3 months–1 year
Liu *et al*.^57^	Diabetic foot	11	VAS score decreased, skin temperature increased, numbness disappeared.	11 (100%)	0	No description	3–24 months
Ding *et al*.^59^	Diabetic foot	12	VAS score decreased, skin temperature and ankle‐brachial index increased, the test results of 10g nylon line improved, CT angiography showed increased collateral arteries, and dorsal foot arteries thickened.	12 (100%)	0	0	5–10 months
Wang *et al*.^63^	The chronic ischemic disease of lower limbs: Diabetic foot (41) Arteriosclerosis obliterans (14) Thromboangiitis obliterans (4)	59	VAS score decreased, skin temperature and ankle‐brachial index increased, CT angiography showed recanalization of the inferior knee anterior tibial artery branches.	54 (91.53%)	4	Skin necrosis (2) Osteomyelitis (1)	8 weeks–25 months Ulcer recurrence (4) The vessel occluded again (4)
Lu et al.^65^	Diabetic foot	45	VAS score decreased, skin temperature and ankle‐brachial index increased, the color ultrasonography revealed collateral branches of arteries in the leg increased, with a clear presentation of the pedis arteries.	45 (100%)	0	Nail infection (1) Deep vein thrombosis in lower limbs (1) Surgical incision infection (1) Pulmonary infection (1)	12–14 months ulcer recurrence (3)
Wang et al.^67^	The chronic ischemic disease of lower limbs: Diabetic foot (25) Arteriosclerosis obliterans (2) Thromboangiitis obliterans (1)	28	Resting pain relieved, the ulcers healed.	25 (89.29%)	1	Needle reaction of external fixator (not infection) (21) Pulmonary infection (1) Acute lower extremity vascular embolism (1) Secondary fracture (1)	5–27 months
Zhao *et al*.^72^	Diabetic foot	32	VAS score decreased, skin temperature increased, the blood flow velocity of dorsal foot artery increased, total peroneal nerve sensation and motor conduction velocity improved, peripheral blood VEGF and PDGF level increased.	32 (100%)	0	Secondary fracture (1)	4–9 months Ulcer recurrence (1)
Xu *et al*.^73^	Thromboangiitis obliterans	35	resting pain and intermittent claudication relieved, skin temperature increased, tissue necrosis decreased, angiography revealed numerous new arterioles extending into the muscle and subcutaneous tissue, pathological examination showed obvious division and hyperplasia of vascular endothelial cells and smooth intima of arterioles.	32 (91.43%)	3	Cerebral fat embolism (1) Skin necrosis (2) Nail infection (4)	2–6 years
Hu *et al*.^74^	Thromboangiitis obliterans	66	VAS score decreased, skin temperature and toe oxygen saturation increased, and angiography revealed new arterioles emerging below the surgical site.	64 (96.97%)	0	No description	2 months
Qin *et al*.^77^	Diabetic foot	19	VAS score decreased, skin temperature and ankle‐brachial index increased insignificantly.	9 (47.37%)	2	Secondary fracture (2) Nail infection (5) The tibia exposed (1)	12–16 months Ulcer recurrence (2)
Yang *et al*.^80^	Thromboangiitis obliterans	20	VAS score and claudication distance decreased, skin temperature and ankle‐brachial index increased.	18 (90%)	2	Ischemia aggravated (1) Osteotomy infection (1)	14–69 months
